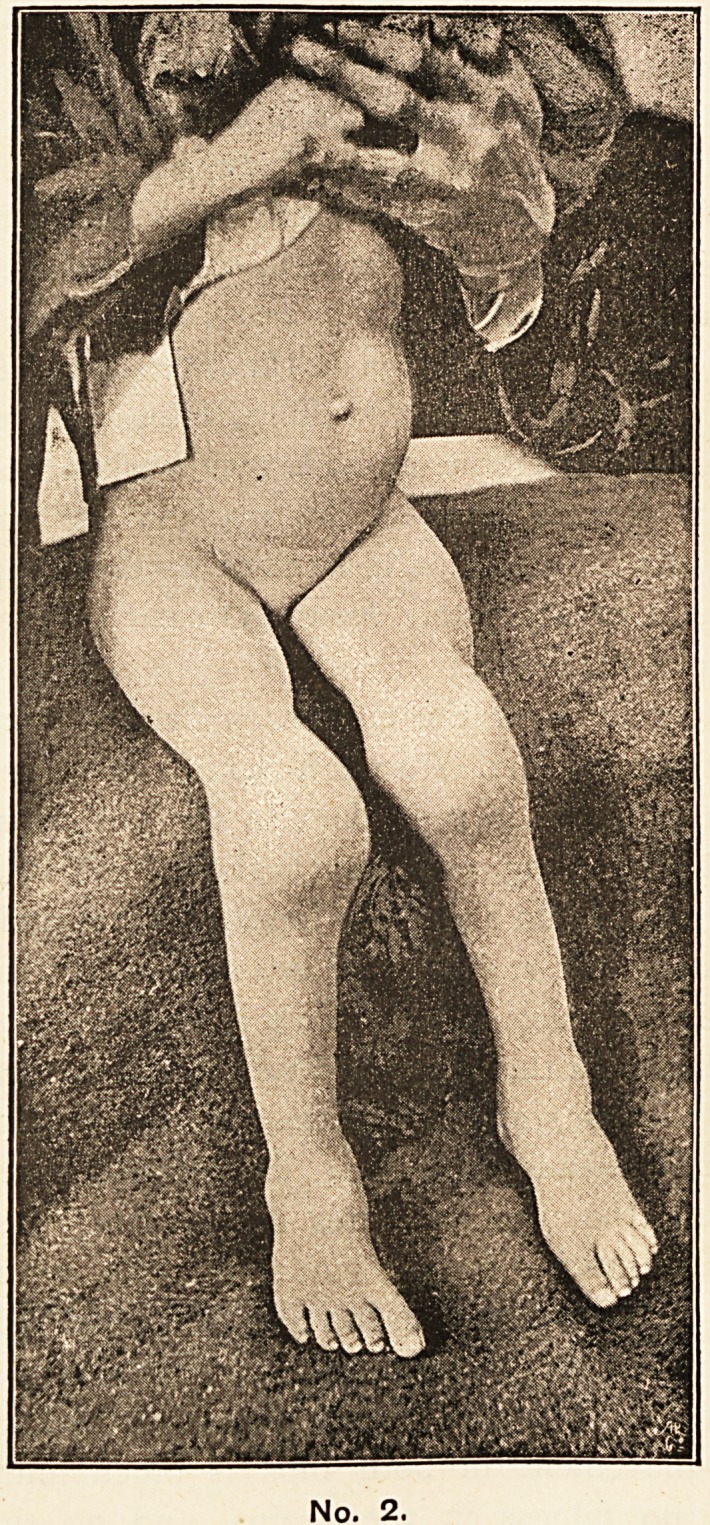# A Case of Multiple Symmetrical Joint Lesions

**Published:** 1892-09

**Authors:** Charles A. Morton

**Affiliations:** Registrar, Bristol General Hospital; Surgical Registrar and Pathologist, Bristol Hospital for Sick Children and Women


### A CASE OF MULTIPLE SYMMETRICAL JOINT LESIONS.

BY

Charles A. Morton, F.R.C.S. Eng.,

*Registrar, Bristol General Hospital; Surgical Registrar and Pathologist, Bristol Hospital for Sick Children and Women.*

---

The following case seems of sufficient interest to be recorded. Both ankles, knees, and elbows are affected, and the back of one carpus. The child, A.H., aged five years, was in the Children's Hospital under the care of Mr. Norton, to whose kindness I am indebted for permission to publish the case. I have had photograph No. 1 taken to show the multiple joint swellings, and the swelling over the left carpus; photograph No. 2 shows the knees on a larger scale.

The knee-joints are seen in the photograph to be much enlarged. On first examining these joints it seems as if the enlargement were in the ends of the bones, particularly the inner condyle, but this is really not so. The swelling consists of pulpy tissue taking the outline of the synovial membrane, with a fluctuating area outside the left ligamentum patellæ. It is the marked atrophy of the thigh muscles just above the knees which makes the end of the bones seem enlarged. So marked is this that measurement around the thigh here only gives six inches, whereas measurement at the same level round the thigh of a well-nourished (but not fat) child only three years of age, gives seven and a half inches. This gives

No. 1.

No. 2.

an *apparent* enlargement of the ends of the bones of nearly two inches. The marked prominence of the inner condyle is, of course, only an exaggeration of the normal. Contraction of the hamstrings limits extension, but the joints can be easily flexed, and there is a marked absence of pain on movement or pressure. A coarse, clicking grating is sometimes felt in them on movement. There is no free fluid in either.

Both ankles are affected in the same way. Here the doughy character of the swelling, filling up the interspaces around the bone-ends, is very marked, and actual bulging is present between the malleoli and the tendo Achillis. As in the knees, movement is perfectly painless, and here it is absolutely free. Occasionally a coarse grating can be felt.

The condition of the elbows on first examination is rather a puzzling one. The soft semi-fluctuating swelling does not take the form it does in the ankles, rounding down all the bony outlines of the joint. It is most marked behind and above the inner condyles and around the head of the radius. It is softer here than in the ankles—in fact, almost fluctuating. Movements of flexion and extension in the joints, and rotation of the radius, are free and painless, except that the arms are kept pronated and cannot be fully supinated. In both, on attempts at supination, the pronator teres is felt as a tense band. The coarse grating is more marked in the left elbow on rotation of the radius than in any other joint. There was at one time some painless fluctuating swelling over the back of the left carpus, which has almost disappeared.

The movement in both hips is much limited, but there is no fulness in the groins, and no thickening around the trochanters.

The child has been under observation for six months. She was brought from a country workhouse infirmary. Since she came, her general condition has greatly improved, but the ankle-joints have become more swollen. The medical officer of the workhouse can give no further information than that she was admitted there in a half-starved, emaciated condition, but quickly began to improve with more food and cod-liver oil. During the time she has been in the Children's Hospital the temperature has always been normal. All the internal organs and the urine are normal. There is no characteristic change in the skull.

The question as to the nature of these multiple joint lesions is of some interest. Are they tubercular or syphilitic? Are they the syphilitic form of joint disease described by Richet under the name of "Syphilitic pseudo-white swelling," a form which Mr. Hutchinson, jun. says may imitate closely the more common strumous arthritis.[1] The following features seem to me to point to their being of this nature. 1. The multiple, symmetrical form. 2. The perfect freedom and painlessness of movement.[2] 3. The coarse grating in the joints, quite unlike the grating of eroded tubercular joint surfaces, and perfectly painless, possibly due to the presence of those pits in the articular surfaces found in this kind of syphilitic joint. 4. The fact that all the joints are in the same stage at the same time; in none has the disease gone on to the formation of pus. At any rate, no external signs of abscess are present.

On the other hand, the very soft character of the

[1] *Brit. Med. Jour.*, April 16, 1892.

[2] Greater mobility and freedom from pain on manipulation than even in early tubercular synovial swelling.

swelling—a swelling which in parts is pulpy (in the ankles wholly so) and in parts fluctuating, without evidence of free fluid in the joints—seems to me more like ordinary tubercular pulpy joint lesions. The absence in the thickened tissue of those hard nodules generally present in the "syphilitic pseudo-white swelling" seems to me also rather against their being of a syphilitic nature.

There are no signs of any tubercular disease in the lungs, and no signs of past syphilitic manifestations. The teeth are, of course, only the temporary set, but they are good, and do not tend to crumble away.

For a fortnight after admission she took five minims of liquor hydrargyri perchloridi three times a day, and for the last three months, with ½-grain doses of iodide of potassium. There has been no improvement in the condition of the joints, but she has gained much flesh, and has so altered for the better in appearance since admission that she would hardly be recognised as the same child.

Mr. Battle has recorded[3] three cases of multiple tubercular joint lesions, but they are quite unlike this case, as they all began in the ends of the bones and involved the joints secondarily. The first case is certainly not tubercular joint disease, and was apparently a case of necrosis of the femur with secondary disease in the hip and knee; in the second there was disease of the knee, associated with caries of the head of the tibia, and then the hip and ankle were affected with suppurative arthritis and a sequestrum was removed from the hip; in the third case there was disease of one hip and one elbow.

[1] *Lancet*, 1889, vol. i., p. 977.

## Figures and Tables

**No. 1. f1:**
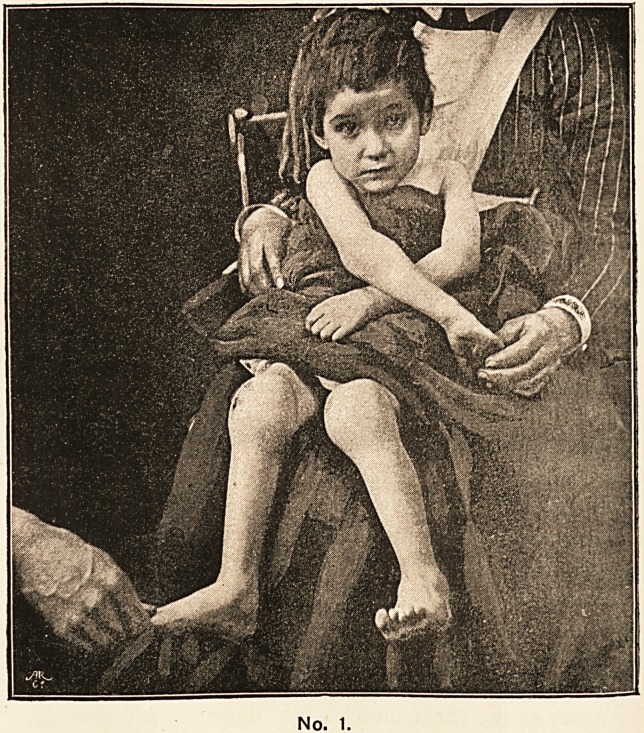


**No. 2. f2:**